# Amplicon sequencing with internal standards yields accurate picocyanobacteria cell abundances as validated with flow cytometry

**DOI:** 10.1093/ismeco/ycae115

**Published:** 2024-09-25

**Authors:** Alexandra E Jones-Kellett, Jesse C McNichol, Yubin Raut, Kelsy R Cain, François Ribalet, E Virginia Armbrust, Michael J Follows, Jed A Fuhrman

**Affiliations:** Department of Earth, Atmospheric, and Planetary Sciences, Massachusetts Institute of Technology, Cambridge, MA 02139, United States; Biology Department, Woods Hole Oceanographic Institution, Woods Hole, MA 02543, United States; Department of Biological Sciences, University of Southern California, Los Angeles, CA 90007, United States; Biology Department, St. Francis Xavier University, Antigonish, NS B2G 2W5, Canada; Department of Earth, Atmospheric, and Planetary Sciences, Massachusetts Institute of Technology, Cambridge, MA 02139, United States; Department of Biological Sciences, University of Southern California, Los Angeles, CA 90007, United States; School of Oceanography, University of Washington, Seattle, WA 98195, United States; School of Oceanography, University of Washington, Seattle, WA 98195, United States; School of Oceanography, University of Washington, Seattle, WA 98195, United States; Department of Earth, Atmospheric, and Planetary Sciences, Massachusetts Institute of Technology, Cambridge, MA 02139, United States; Department of Biological Sciences, University of Southern California, Los Angeles, CA 90007, United States

**Keywords:** internal genomic standards, spike-in, absolute abundance, picocyanobacteria, Prochlorococcus, Synechococcus, 16S rRNA, amplicon sequencing, flow cytometry, North Pacific Ocean

## Abstract

To understand ecosystem state and function, marine microbial ecologists seek measurements of organismal abundance and diversity at high taxonomic resolution. Conventional flow cytometry accurately estimates microbial cell abundance but only discerns broad groups with distinct optical properties. While amplicon sequencing resolves more comprehensive diversity within microbiomes, it typically only provides relative organismal abundances within samples, not absolute abundance changes. Internal genomic standards offer a solution for absolute amplicon-based measures. Here, we spiked genomic standards into plankton samples from surface seawater, gathered at 46-km intervals along a cruise transect spanning the southern California Current System and the oligotrophic North Pacific Subtropical Gyre. This enabled evaluation of the absolute volumetric gene copy abundances of 16S rRNA amplicon sequence variants (amplified with 515Y-926R universal primers, quantitatively validated with mock communities) and cell abundances of picocyanobacteria with known genomic 16S copy numbers. Comparison of amplicon-derived cell abundances of *Prochlorococcus* and *Synechococcus* with flow cytometry data from nearby locations yielded nearly identical results (slope = 1.01; Pearson’s *r* = 0.9942). Our findings show that this amplicon sequencing protocol combined with genomic internal standards accurately measures absolute cell counts of marine picocyanobacteria in complex field samples. By extension, we expect this approach to reasonably estimate volumetric gene copies for other amplified taxa in these samples.

Amplicon sequencing reveals planktonic community structure in unprecedented detail [1]. Yet, limitations remain, as studies rely on the interpretation of organismal abundances relative to the whole community due to an unknown PCR amplification factor [2]. Minor changes in community composition can have dramatic effects when comparing relative abundances between samples. For example, if the abundance of one taxon changes while others remain constant, the relative abundance shifts for all taxa. Thus, fluctuations in relative abundance do not necessarily indicate changes in absolute abundance. It is also difficult to independently validate amplicon-based estimates since other sampling tools, such as flow cytometry (FCM), measure absolute abundance for organisms with distinct optical properties (e.g. picocyanobacteria), but with low taxonomic resolution.

By spiking samples with a known quantity of DNA of non-marine organisms (i.e. internal genomic standards), the PCR amplification factor is unveiled, enabling the estimation of volumetric gene copies for all amplicon sequence variants (ASVs) [3]. Recently, studies have utilized internal standards to quantify marine community composition [4–8]. Lin *et al.* [5] compared internal standard-corrected total bacterial abundance with FCM, but their comparisons were not taxon-specific. None of the cited studies collected technical replicates to quantify methodological error. Here, we spiked microbial samples collected in triplicate every 46 km along a North Pacific Ocean transect with internal genomic standards. We used an amplicon method that accurately evaluates relative gene copy abundance [9–11] and compared taxon-specific estimates of picocyanobacteria with direct cell counts obtained by FCM. These independent methods yielded nearly identical results, validating their quantitative nature.

We filtered surface seawater samples in triplicate at 65 sites from the underway system of the *R/V Thompson* during the November 2021 SCOPE Gradients 4 cruise (Fig. 1; Text S1A). We pre-aliquoted 20 $\mathrm{\mu}$l (~4 ng) for each of three genomic standards (*Blautia producta*, *Deinococcus radiodurans*, *Thermus thermophilus*) for each sample, to make up ~1% of the total DNA [6]. Then, we added the standards to the lysis buffer before DNA extraction [13]. We amplified the DNA with the 515Y (59-GTGYCAGCMGCCGCGGTAA) and 926R (59-CCGYCAATTYMTTTRAGTTT) primers [9]. Text S2 details the protocols for DNA extraction and PCR. HiSeq RapidRun technology (2 × 250 bp) was used for sequencing, and we followed a pipeline for ASV identification and taxonomic classification that was specifically developed for the primer set [10, 11] (Text S3).

We obtained nine volumetric 16S rRNA gene copy count estimates from three technical replicates and three internal standards for each sample site and ASV. The gene copies for the taxon *i* in the sample *j* estimated from the genomic standard *s* is


$$ {\mathrm{ASV}}_{ij}=\frac{R_{ij}\cdotp{C}_s}{R_{sj}\cdotp{V}_j} $$

where ${R}_{ij}$ is the number of reads, ${C}_s$ is the number of 16S rRNA gene copies of $s$ (known a priori), ${R}_{sj}$ is the number of reads of $s$, and ${V}_j$ is the volume of filtered seawater [5] (Text S4). We estimated taxon-specific abundances of picocyanobacteria assuming *Prochlorococcus* cells contain one 16S rRNA gene copy per cell [14] and *Synechococcus* contains two [15]. Table S1 lists the unique ASV IDs categorized as *Prochlorococcus* or *Synechococcus* with non-zero abundance. The absolute abundance is defined for each ASV as the mean of the nine estimates. 95% of *Prochlorococcus* cell abundance estimates had a percent error <28.6% relative to the sample mean, and <31.7% for *Synechococcus* (Text S5).

**Figure 1:**
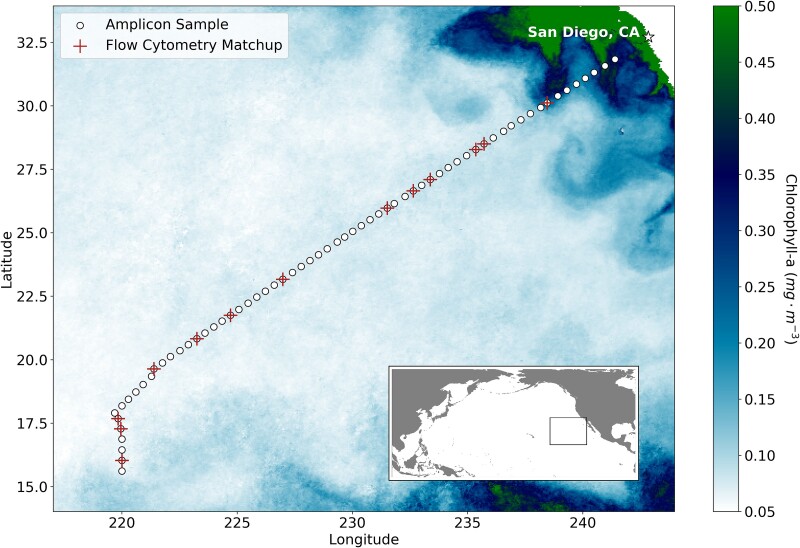
Sample sites overlaid on the mean November 2021 OC-CCI satellite chlorophyll-*a* (a proxy for phytoplankton biomass) [12]. Amplicon samples (*N* = 65) are represented by white dots, and FCM sample matchups within a 20 km distance and 6-h window (*N* = 13) are labeled with red crosses.

We measured *Prochlorococcus* and *Synechococcus* cell abundances using a BD Influx flow cytometer three times daily, in triplicate, from the same underway system used to obtain the amplicon samples [16] (Text S1B). In Figs 1 and 2, we compared the amplicon estimates with FCM samples collected within 20 km and a 6-h window, providing 13 matchups (Table S3). The FCM abundances are defined as the means of the triplicate estimates, for which 95% of *Prochlorococcus* estimates had a percent error <10.8% relative to the sample mean, and <15.7% for *Synechococcus*.

**Figure 2:**
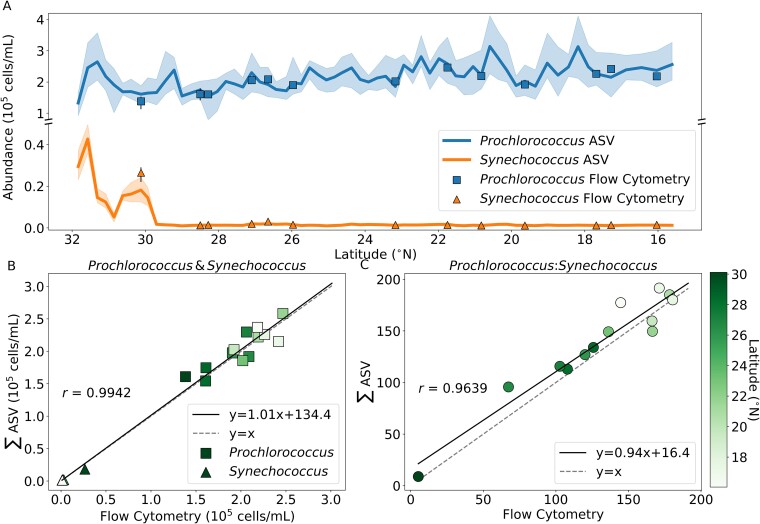
Comparison of amplicon and FCM-estimated cell counts of *Prochlorococcus* and *Synechococcus*. Sample matchups (*N* = 13) were collected within 20 km and a 6-h window. (A) The solid lines show the mean ASV estimated cell count and the shadows show the range in estimated values. The squares show the mean FCM estimated cell count at the matchup ASV sample site, and the vertical black lines show the range in measurements. (B) Linear regression of the amplicon versus FCM *Prochlorococcus* and *Synechococcus* cell counts (slope = 1.01; Pearson’s *r* = 0.9942). The data points are colored by latitude. Linear regressions of *Prochlorococcus* and *Synechococcus* separately are in supplementary Fig. S6. (C) Same as (B) but the ratio of *Prochlorococcus* to *Synechococcus* cell abundances (slope = 0.94; Pearson’s *r* = 0.9639).

The amplicon and FCM-derived picocyanobacteria cell abundance estimates are linearly correlated with slope 1.01 (Pearson’s *r* = 0.9942; Fig. 2B). The *Prochlorococcus* to *Synechococcus* ratios also linearly correlate with slope 0.94 (Pearson’s *r* = 0.9639; Fig. 2C). We tested the sensitivity of these results to the geographic distance criterion of 20 km. A more lenient threshold of 30 km yielded more pairings (N = 22), but the *Prochlorococcus* to *Synechococcus* ratio was less strongly correlated (slope = 0.78, Pearson’s *r* = 0.8793; Fig. S1). Reducing the distance to 10 km provided eight matchups, and the ratio correlation improved (slope = 0.98, Pearson’s *r* = 0.9644; Fig. S2). Thus, as expected, geographically closer samples generate more similar estimates of the ratios of *Prochlorococcus* to *Synechococcus*. However, the distance criterion did not greatly affect the cell abundance correlations (Figs S1 and S2).

These results demonstrate the effectiveness of using high-throughput amplicon sequencing and internal standards to quantify absolute ASV abundances. We observed one-to-one correlations by comparing the amplicon-estimated abundances of picocyanobacteria across sample sites with FCM data. While most cell counting methods categorize cells by functional group or size, amplicon sequencing offers a much finer taxonomic resolution. This is exemplified in our ASV dataset, which revealed a latitudinal transition in the dominant ecotype of *Prochlorococcus* (Fig. S3), a detail undetected at the broader group level or from FCM alone. The patterns of absolute and relative abundances at the ASV, ecotype, and group levels can differ substantially (Figs S3–S5), highlighting the advantage of internal standards to interpret changes in planktonic community structure.

Determining cell abundances of non-picocyanobacteria presents challenges because many taxa have multiple rRNA gene copies per genome and the copy number is often unknown for uncultivated taxa [17, 18]. Nevertheless, internal standard spike-ins are likely to yield good volumetric gene copy estimates for all amplified taxa in these samples with our protocol, given that the primers and bioinformatic pipeline accurately estimate relative abundances for prokaryotes and eukaryotes in mock communities [9, 11]. Further, the primers match >95% of rRNA gene sequences in marine metagenomes [10]. Since other popular primers are less accurate for relative abundances when tested with mock communities [9, 11], we expect they may return less exact absolute abundances (Text S6). Other taxon-specific abundances derived from these methods could be validated with, e.g. quantitative PCR [19], CARD-FISH [20], or imagery [21]. Analogous benchmarking should be conducted with a well-studied taxon to verify that results are similarly quantitative for application to other ecosystems. We encourage future studies to incorporate internal standards in their amplicon sequencing pipelines to measure absolute changes in volumetric 16S (and 18S) rRNA gene copies across samples and quantitatively capture the relationships between organisms and their environment.

## Acknowledgements

Thank you to the crew of the *R/V Thompson* (TN-397) and the SCOPE Operational Staff, Brandon Brenes and Timothy Burrell, for making the SCOPE Gradients 4 cruise a success. Zoe Finkel, Andrew Irwin, and Ruby Hu were instrumental in planning the field campaign logistics. We thank Niall McGinty, Brian Beardsall, and Sing-How Tuo for their help with onboard sample collection. Kema Malki ordered and prepared supplies for the cruise. Laura Furtado provided laboratory assistance and Bruce Yanpui Chan aided in the sample preparation for PCR. Colette Fletcher-Hoppe helped to prepare the 16S/18S merged ASV table. We thank five anonymous reviewers and the editors for their insightful comments and questions that improved the presentation of this manuscript.

## Author contributions

A.E.J.K., J.C.M., Y.R., M.J.F., and J.A.F. contributed to the conceptualization of this manuscript. A.E.J.K., J.C.M., M.J.F., and J.A.F. designed the amplicon methodologies. A.E.J.K. collected and filtered seawater samples, performed the formal analysis, and generated the figures. A.E.J.K. and J.M. conducted the DNA extractions. A.E.J.K., J.C.M., and Y.R. prepared the samples for PCR and sequencing. J.C.M. ran the software for ASV identification and taxonomic classification. K.R.C., F.R., and E.V.A. designed the FCM methodologies. K.R.C. collected and processed the shipboard FCM samples. F.R. designed the software for FCM data analysis. M.J.F., J.A.F., and E.V.A. acquired funding that supported this project. A.E.J.K. wrote the original draft and all authors reviewed and edited the manuscript.

## Conflicts of interest

None declared.

## Funding

We received generous funding from the Simons Foundation that supported this work (SCOPE Awards 329108, M.J.F. and 723795, E.V.A.; CBIOMES Awards 549931, M.J.F. and 549943, J.A.F.).

## Data availability

The taxonomic classification software for the 515Y/926R primers is available at github.com/jcmcnch/eASV-pipeline-for-515Y-926R [10]. The Python software used to calculate the internal standards is available at github.com/lexi-jones/internal_std_correction. The sequences are available at NCBI Sequence Read Archive (BioProject ID: PRJNA1079727). The SCOPE Gradients 4 cruise BD influx FCM data is distributed by Simon’s CMAP and available at simonscmap.com/catalog/datasets/Influx_Underway_Gradients_2021 [16]. Picocyanobacteria cell abundances from FCM were estimated using the FCSplankton R package available at https://github.com/fribalet/FCSplankton. The chlorophyll-*a* data in Fig. 1 is from the OC-CCI Version 6.0 daily product, distributed by the European Space Agency at oceancolour.org [12]. The global map overlay was generated with the geopandas and cartopy Python packages. The linear regression statistics were calculated with scipy.stats.linregress. All figures were generated with Matplotlib 3.3.4.

## Supplementary Material

ISME_Comm_Supp_Final_ycae115
