# Supplementary material for: Amplicon sequencing with internal standards yields accurate picocyanobacteria cell abundances as validated with flow cytometry
Source: ISME Commun. 2024 Sep 25;4(1):ycae115. doi: 10.1093/ismeco/ycae115 (PMC11459381; doi:10.1093/ismeco/ycae115)
Supplement: ISME_Comm_Supp_Final_ycae115 [file isme_comm_supp_final_ycae115.pdf]

Supplemental Material for the manuscript "*Amplicon Sequencing with Internal Standards Yields Accurate Picocyanobacteria Cell Abundances as Validated with Flow Cytometry*" by Alexandra E. Jones-Kellett[1,2,+], Jesse C. McNichol[3,4], Yubin Raut[1,3], Kelsy R. Cain[5], François Ribalet[5], E. Virginia Armbrust[5], Michael J. Follows[1], and Jed A. Fuhrman[3]

[1]Department of Earth, Atmospheric, and Planetary Sciences, Massachusetts Institute of Technology, Cambridge, MA, USA

[2]Biology Department, Woods Hole Oceanographic Institution, Woods Hole, MA, USA

[3]Department of Biological Sciences, University of Southern California, Los Angeles, CA, USA

[4]Biology Department, St. Francis Xavier University, Antigonish, NS, Canada

[5]School of Oceanography, University of Washington, Seattle, WA, 98195, USA

[+]Corresponding author (email: jonesae@mit.edu)

Contents

I. Texts S1 – S6

II. Figures S1 – S6

III. Tables S1 – S3

IV. References

I. Texts S1 – S6

Text S1. Shipboard Sample Methods

A. Amplicon Samples

We collected more than three liters of water from the underway seawater system at each sampling site in one carboy, briefly mixed it, and then separated the seawater into three 1L bottles to serve as technical replicates. We filtered each 1L replicate through 0.22μm Sterivex-GV filters (EMD Millipore; SVGVL10RC) with a peristaltic pump. Filters were sealed with Luer lock plug caps (MRO Supplies; 51525K333, 51525K334) and frozen at -80°C. In total, samples were collected at 65 locations, but not all samples had a comparable flow cytometry measurement matchup.

B. Flow Cytometry

We collected live samples into 250mL amber bottles for flow cytometry (FCM) three times daily, in triplicate, from the underway seawater system. Samples were prefiltered with a 100µm Nitex mesh. We made the measurements on a BD Influx flow cytometer (BD, Franklin Lakes, NJ, USA), which was equipped with two 10% neutral density filters, a 488nm laser, and the small particle detector option. Data collection was triggered by forward light scatter. For each sample, we added 1μm beads (Invitrogen, Waltham, MA, USA) that were used as an internal standard to a final 1:1x10^7 dilution (5μL of 1:100,000 stock to 495μL of sample). We identified picocyanobacterial cells by sequentially gating using the FCSplankton package in R (https://github.com/fribalet/FCSplankton). Specifically, we distinguished *Synechococcus* cells by their forward scatter and 580/30 nm emission characteristic, then we identified *Prochlorococcus* cells by their forward scatter and 692/40 nm emission. The FCM data is publicly available on Zenodo and Simon's CMAP [1].

In total, we used 23 FCM samples in this study located within 30km and a 6-hour window of an amplicon sample (Table S3). Two FCM samples met these criteria for the same amplicon sample and

were treated as replicates for N=22 matchups. The main text uses a stricter distance criterion of 20km, resulting in 13 matchups. Figures S1 and S2 show how altering this distance criterion affects the results.

Text S2. DNA Extraction and PCR Amplification

A. Sterivex Filter Membrane Removal

We sterilized the benchtop with 70% ethanol, followed by distilled water [2]. In an RNAse/DNAse-free low-bind 2mL tube (Eppendorf) we aliquoted 1.5mL of lysis buffer (composed of 0.4 mol NaCl/L, 0.75 mol sucrose/L, 0.02 mol EDTA/L, and 0.05 mol TrisHCl/L dissolved in molecular-grade water and adjusted to pH 9 with molecular grade NaOH) and 20μL (~4ng) of three genomic standards (*Blautia producta*, *Deinococcus radiodurans*, *Thermus thermophilus*) for each sample [3] using an Eppendorf XStream automatic repeating pipettor with 0.5mL sterile DNA/RNA-free combitips.

After preparation of the lysis buffer and internal standards, we removed the samples from -80°C and kept them on ice. We used flame-sterilized pliers to crack open the Sterivex cartridges and extract the filters within. We used flame-sterilized razor blades and tweezers to cut the filters from the interior of the cartridge, fold them, and place them in the lysis buffer. The filter membranes were removed next to a Bunsen burner and on a clean Petri dish to prevent contamination.

B. Cell Lysis

We followed protocols adapted from [4-6] for cell lysis and DNA recovery because they have higher extraction yields than classic phenol/chloroform DNA extraction protocols. We added 30μL of lysozyme (1 mg/mL solution) to the 2 mL tubes and then incubated samples for 30 minutes at 37°C. After incubation, we added 180μL of sodium dodecyl sulfate (10% solution) and 0.25g of pre-

combusted beads (0.1mm; Biospec 11079101). We added a bead-beating step to reduce the possibility of extraction biases against organisms with a harder cell exterior (organic or mineral) that may have been resistant to the lysis techniques. We bead-beat the samples for 2 minutes on maximum speed with a VWR Analog Vortex Mixer and then added 1μL of proteinase-K (20 mg/mL; NEB P8107S). We incubated the samples overnight (>=12 hours) at 55°C. After incubation, we transferred 400μL of the supernatant to a 5mL tube and added 700μL of TE buffer (high-EDTA: 10mM Tris-HCl, 1mM EDTA, pH 7.4).

C. DNA Recovery

We added 5μL of Linear Acrylamide (5mg/mL) to the samples as a co-precipitant [6]. We added 350μL of 3M Sodium Acetate and 3.5mL of ethanol (100%). We inverted the tubes to mix phases before incubating for 1 hour at -20°C. Next, we centrifuged the samples at 20,000×g for 20 minutes at 4°C to pellet the DNA. We slowly drew off the supernatant with a pipette until only the DNA pellet remained in the tube. We added 500μL of 100% ice-cold ethanol on top of the pellet to wash off contaminating substances (e.g. pigments, digested proteins, lipids, etc.). Then with ethanol, we centrifuged the pellet again using the same settings as before. We slowly drew the ethanol off the DNA pellet. We left the pellets to air dry for 30 minutes in a biosafety cabinet to evaporate any remaining ethanol. We resuspended the DNA pellet in 50μL of TE buffer (low-EDTA: 10mM Tris-HCl, 0.1mM EDTA, pH 8.0; Quality Biological) and utilized the TE droplet to gather any remaining DNA on the sides of the tube from the centrifugation. We left the pellet to dissolve for one hour at 4°C, then stored the DNA samples at -80°C until the preparation for PCR.

D. PCR

The wet lab procedures followed are available at doi.org/10.17504/protocols.io.vb7e2rn, except we used the GoTaq master mix (Promega, M5132/5133; Lot #0000520348) for amplification.

Text S3. Sequencing & Annotation

Samples were sequenced at Tufts University Medical School using the HiSeq RapidRun technology (2x250 bp). We manually demultiplexed the samples using the software packages bcl2fastq and cutadapt (doi:10.14806/ej.17.1.200) following the steps at github.com/jcmcnch/demux-notes. We used a software pipeline developed for the primer set 515Y/926R for the Amplicon Sequence Variant (ASV) identification and taxonomic classification, available at github.com/jcmcnch/eASV-pipeline-for-515Y-926R (version:qiime2-2022.2-DADA2-SILVA138.1-PR2_4.14.0) [7,8]. This software splits the data into 16S and 18S rRNA and denoises them separately. There is a known PCR bias against 18S sequences that needed to be corrected for [8]. We used an Agilent 2100 Bioanalyzer to quantify concentrations of amplicons in the sequencing pool, and the correction factor (=2.09) was derived by comparing the 16S:18S ratio of molarity. This correction factor was applied along with sample-specific adjustments to account for sequence loss during QC steps to merge the 16S and 18S reads into a single ASV table using a custom R script (github.com/fletchec99/normalizing_16S_18S_tags).

We categorized all taxa with the key string "*Synechoccales*" as picocyanobacteria. We used ProPortal to assign a subset of the picocyanobacteria ASVs more specifically as *Prochlorococcus* or *Synechococcus* [9]. Furthermore, ProPortal assigned *Prochlorococcus* ecotypes when known. Of the remaining picocyanobacteria ASVs not assigned by ProPortal, we categorized them as *Prochloroccocus* or *Synechococcus* if they had the corresponding string in their taxonomic assignment. We excluded ASVs from this study if their assignment beyond picocyanobacteria was ambiguous. 153 ASVs with non-zero

abundance were categorized as *Prochlorococcus*, and 27 as *Synechococcus*. Table S1 includes the ASVs used in this study and their taxonomic assignments.

Text S4. ASV Abundance

We calculated the number of 16S rRNA gene copies of the genomic standard ($gDNA$),

$$C_s = \frac{gDNA_m \cdot A \cdot rrn_s}{gDNA_l \cdot F \cdot bp_m},$$

where $gDNA_m$ is the genome mass of the genomic standard, $A = 6.022 \times 10^{23}(mol^{-1})$ is Avogadro's number, rrn_s is the number of 16S rRNA copy numbers per cell in the genomic standard, $gDNA_l$ is the genome length in base pairs of the genomic standard, $F = 10^9(ng \cdot g^{-1})$ is a conversion factor, and $bp_m = 650\ (g \cdot mol^{-1} \cdot bp^{-1})$ is the average weight of a base pair. The genome mass of the genomic standard is

$$gDNA_m = conc_s \cdot V_s,$$

where $V_s = 20\mu L$ is the volume of internal standard spiked in the sample and $conc_s$ is the concentration of the genomic standard.

We measured the concentration of the internal standards with a Pico-Green dsDNA Quant-iT Assay Kit on a Synergy H1 microplate reader (Agilent) using 96-well plates (clear-bottom / black side, Corning #3651) using excitation/emission wavelengths of 485/538nm. We carried out all dilutions of the samples and Pico-Green reagents in low-EDTA TE buffer to prevent DNA degradation. Assays were conducted by first adding samples to 100 µL low-EDTA TE in 96-well plates, followed by adding 100 µL Pico-Green dye diluted to the working concentration with an automatic repeating pipettor. We incubated the samples in the dark for 10 minutes before measurement on the plate reader. The total reaction volume was 200 µL plus 1-10 µL of the sample (depending on the concentrations measured; total volume was always

consistent within each assay). Lambda phage DNA was used as a standard and diluted from a 100 ng/µL stock in low-EDTA TE (always stored at 4°C) immediately before measurement.

Table S2 includes the values underlying C_s for each genomic standard, and the code to calculate the ASV abundances is available at github.com/lexi-jones/internal_std_correction.

Text S5. ASV Abundance Variability

The total variability in the amplicon-derived estimates of *Prochlorococcus* and *Synechococcus* originates from the three technical replicates at each sample site and the three internal standard corrections. The total error is the product of both sources of variability. 95% of *Prochlorococcus* relative abundance estimates had a percent error <10.1% relative to the sample mean, and <19.0% for *Synechococcus*.

Within a sample, the variability of the internal standard correction can be measured by comparing C_s/R_{sj} between each of the three standards *s* in sample *j*, where C_s is the number of 16S rRNA gene copies of the genomic standard and R_{sj} is the number of reads. C_s/R_{sj} for 95% of samples varied by <33.1% when comparing each internal standard and the average variance was 19.4%. This variance will affect the range of absolute abundance estimated for each ASV equally within a sample.

Text S6. 515Y-926R Primer Set

The 515Y-926R primers are accurate when tested with dozens of mock community members. When other popular primers have been tested, they have been considerably less accurate even when the primers perfectly match the mock community members. For example, in Parada et al. (2016) [10], when testing the EMP (515C-806R) primers, the r^2 between observed and expected was 0.53, as opposed to

0.95 with the 515Y-926R primers. For 515C-806R, it improved only to r^2=0.69 when SAR11 and SAR116 clones (each having mismatches) were excluded from the calculations.

Yeh et al. (2021) [8], notably Fig 4B, showed that the commonly used original Stoeck et al. (2010) [11] V4 eukaryote primers were surprisingly inaccurate even with no mock community mismatches. Only perfectly matching mock community members with these V4 primers had a r^2= 0.66, vs r^2=0.97 for 515Y-926R primers (Fig 4B in [8]). When considering all mock members, some of which have mismatches to the 515Y-926R primers, the r^2 values were 0.65 for V4 and 0.76 for 515Y-926R (Fig 4A in [8]).

As long as a primer set is free of major amplification biases, it should perform quantitatively in conjunction with internal standard spike-ins. However, validation with mock communities is first needed to trust a quantitative interpretation. An additional benefit of the 515Y-926R primer set is that it is uniquely appropriate for whole-ecosystem surveys.

II. Figures S1- S6

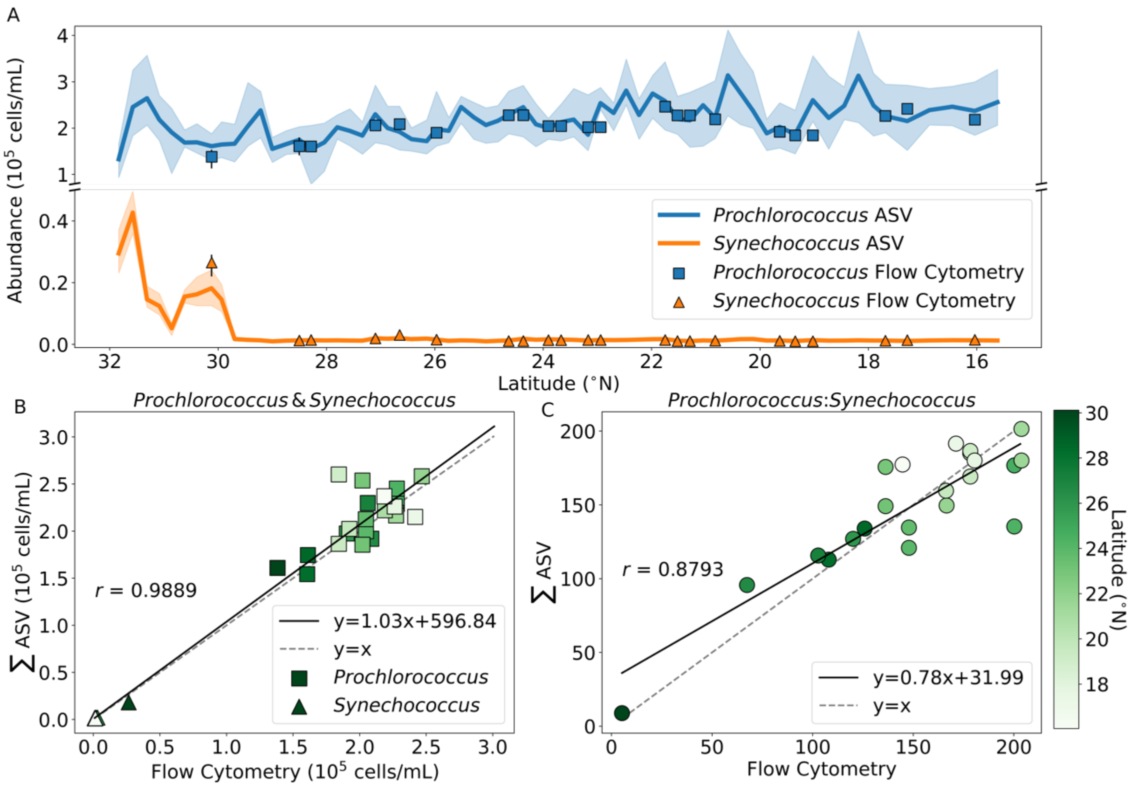

Figure S1. Comparison of amplicon and FCM-estimated cell counts of *Prochlorococcus* and *Synechococcus.* Sample matchups (N=22) were collected within 30 kilometers and a 6-hour window. **A)** The solid lines show the mean ASV estimated cell count and the shadows show the range in estimated values. The squares show the mean FCM estimated cell count at the matchup ASV sample site, and the vertical black lines show the range in measurements. **B)** Linear regression of the amplicon versus FCM *Prochlorococcus* and *Synechococcus* cell counts (slope=1.03; Pearson's r=0.9889). The data points are colored by latitude. **C)** Same as (B) but the ratio of *Prochlorococcus* to *Synechococcus* cell abundances (slope=0.78; Pearson's r=0.8793).

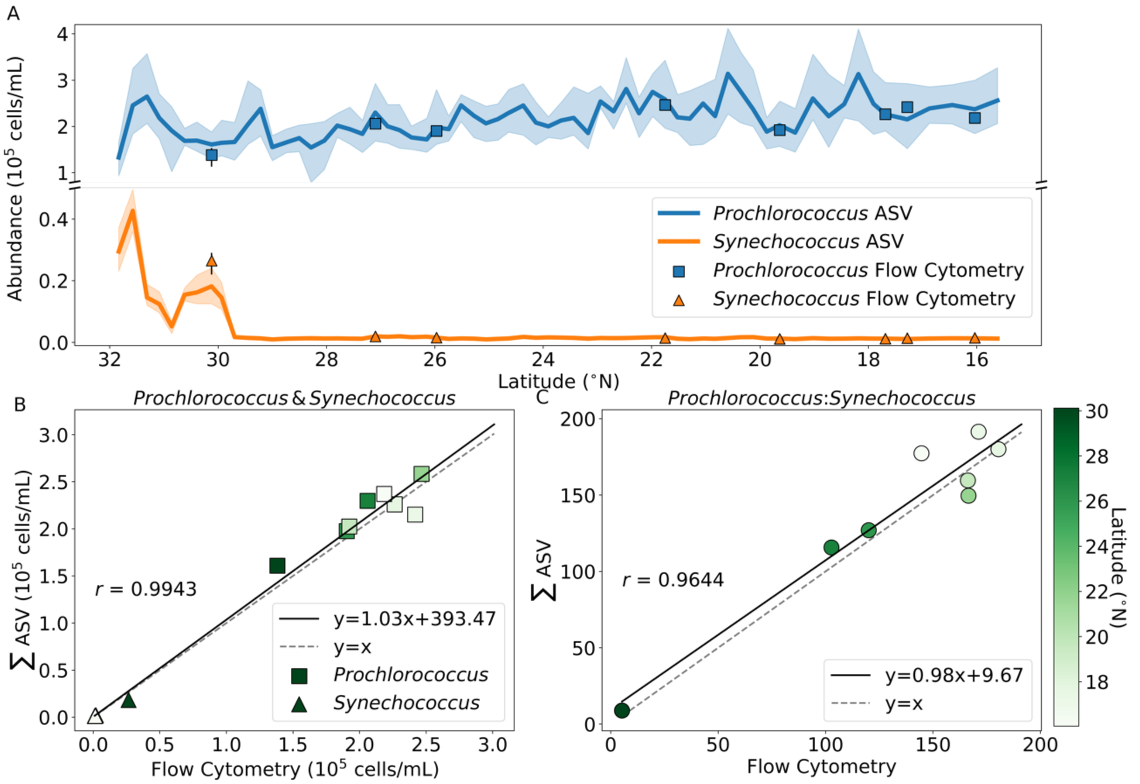

Figure S2. Comparison of amplicon and FCM estimated cell counts of *Prochlorococcus* and *Synechococcus.* Sample matchups (N=8) were collected within 10 kilometers and a 6-hour window. **A)** The solid lines show the mean ASV estimated cell count and the shadows show the range in estimated values. The squares show the mean FCM estimated cell count at the matchup ASV sample site, and the vertical black lines show the range in measurements. **B)** Linear regression of the amplicon versus FCM *Prochlorococcus* and *Synechococcus* cell counts (slope=1.03; Pearson's r=0.9943). The data points are colored by latitude. **C)** Same as (B) but the ratio of *Prochlorococcus* to *Synechococcus* cell abundances (slope=0.98; Pearson's r=0.9644).

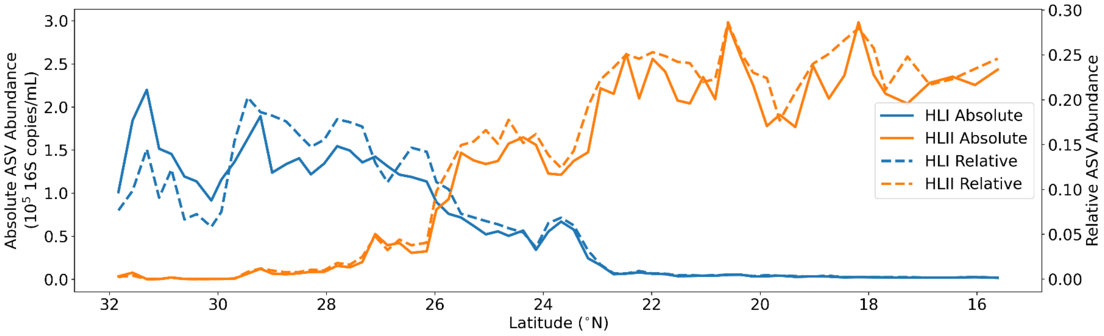

Figure S3. Absolute (solid lines) and relative (dotted lines) abundances of *Prochlorococcus* ecotypes High-Light I (HLI) in blue and High-Light II (HLII) in orange. The dotted lines reflect the ASV abundances relative to all 16S and 18S ASVs (i.e., the whole community).

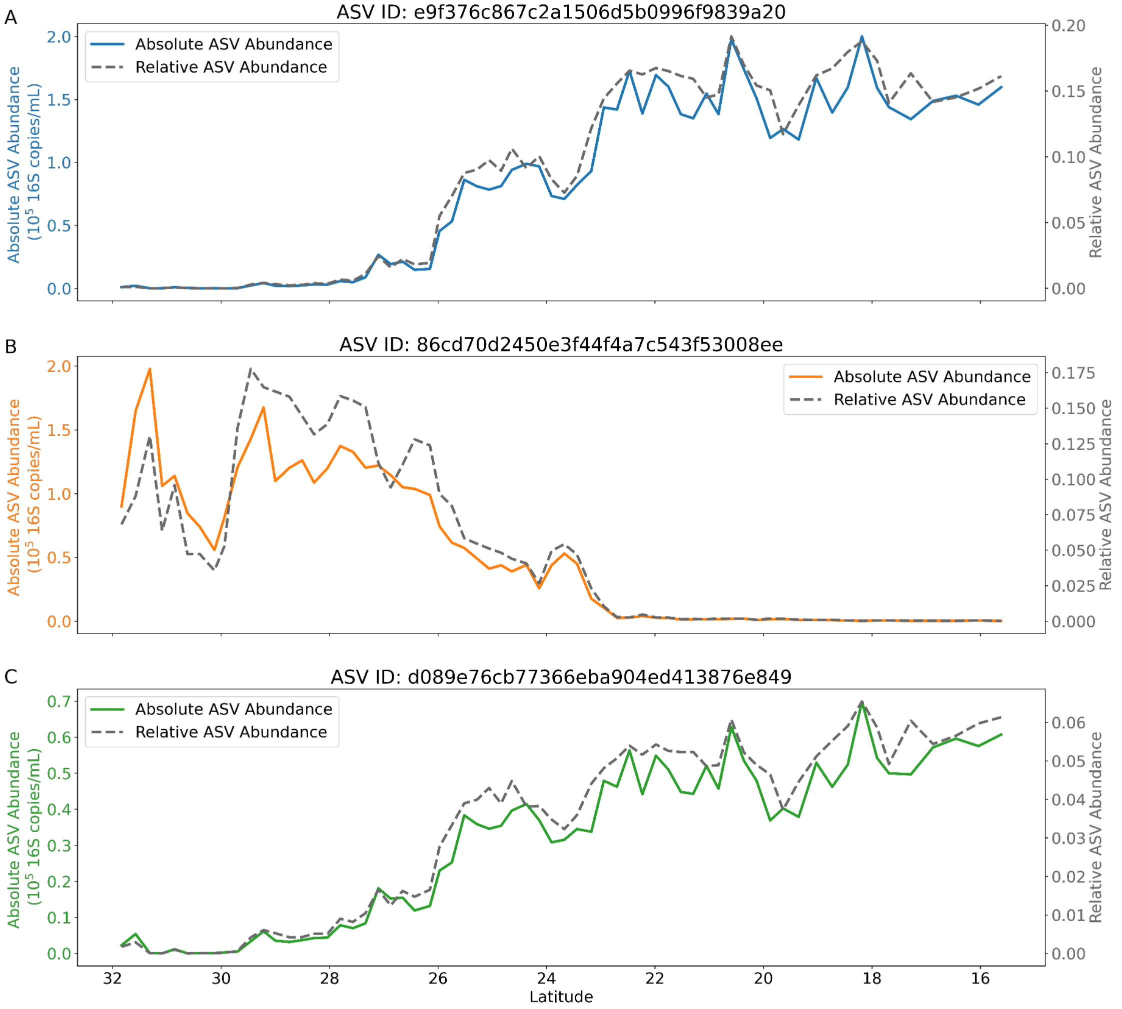

Figure S4. The absolute (colored, solid lines) and relative (grey, dotted lines) abundances of the three most abundant *Prochlorococcus* Amplicon Sequence Variants (ASVs). The grey dotted lines reflect the ASV abundances relative to all 16S and 18S ASVs (i.e., the whole community).

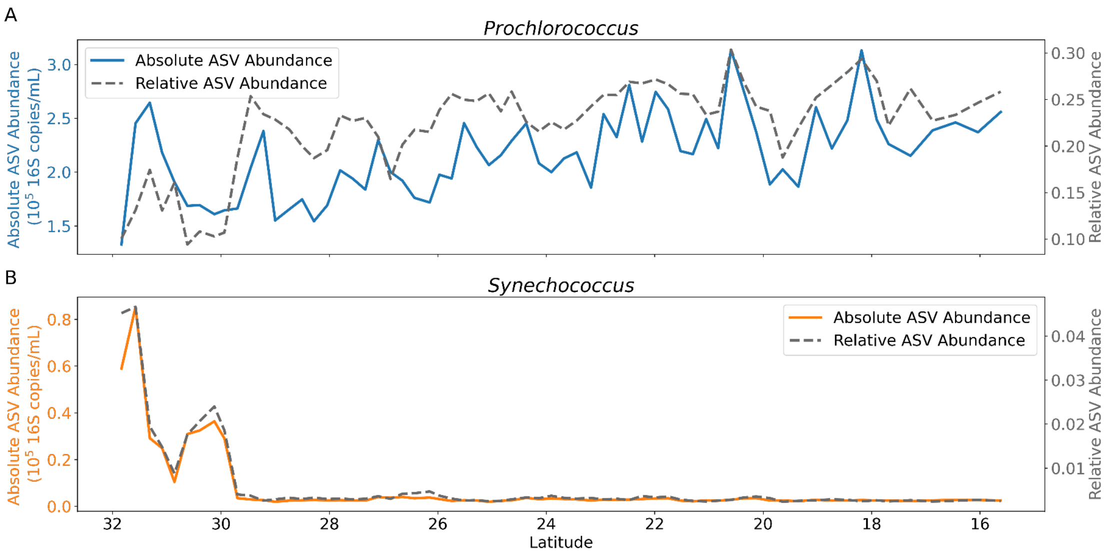

Figure S5. The sum of absolute (colored, solid lines) and relative (grey, dotted lines) abundances of *Prochlorococcus* (A) and *Synechococcus* (B) ASVs. The grey dotted lines reflect the ASV abundances relative to all 16S and 18S ASVs (i.e., the whole community).

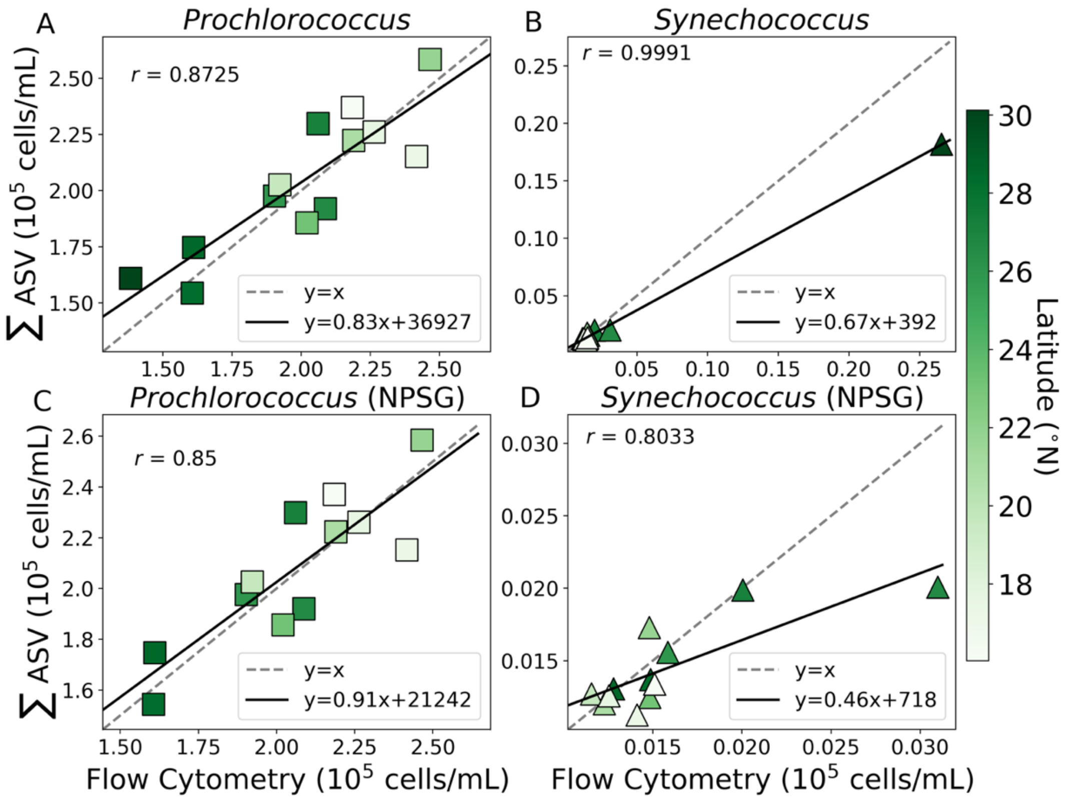

Figure S6. Decomposition of Figure 2B of the main text. A) Amplicon versus FCM estimated abundances of only *Prochlorococcus* (slope=0.83; Pearson's r=0.8725) and B) only *Synechococcus* (slope=0.67; Pearson's r=0.9991). To test the effects of the only sample pairing in the California Current System, we removed that data point for C) *Prochlorococcus* (slope=0.91; Pearson's r=0.85) and D) *Synechococcus* (slope=0.46; Pearson's r=0.8033). Removing the northernmost sample made the slope closer to 1 for *Prochlorococcus* but further for *Synechococcus* because one gyre sample had much higher *Synechococcus* cell counts measured by FCM than the amplicon method. The cause of the outlier data point is unclear.

III. Tables S1 - S3

Table S1. Picocyanobacteria ASV IDs included in the analysis (N=180) and their taxonomic assignments.

ASV ID	Assignment	ProPortal Assignment
**e9f376c867c2a1506d5b0996f9839a20**	Prochlorococcus	Prochlorococcus_HLII
**86cd70d2450e3f44f4a7c543f53008ee**	Prochlorococcus	Prochlorococcus_HLI
**d089e76cb77366eba904ed413876e849**	Prochlorococcus	Prochlorococcus_HLII
**27ca2b8f287c3a7e4d864cc870cf67b7**	Prochlorococcus	Prochlorococcus_unassigned
**7a1011dfbc8b253cb0032a00e74454e9**	Prochlorococcus	Prochlorococcus_HLI
**5cadb906e927ec37f15cd2b07b7d9da3**	Prochlorococcus	Prochlorococcus_HLII
**44369269b45b7e1a562a501cea30045e**	Prochlorococcus	Prochlorococcus_HLII
**7fda097303c92051e93b901f30103dcd**	Prochlorococcus	Prochlorococcus_HL
**31f1c6b0fc2ac518e94e7cadfe7f28a1**	Prochlorococcus	Prochlorococcus_HLII
**8e8c9fdc7c501094f3925d17e4ab91b0**	Prochlorococcus	Prochlorococcus_HL
**912dc151e3ecdf57069e8103e82ca984**	Prochlorococcus	Prochlorococcus_HLII
**ad7a812323880fdbfb5bb08777efac8e**	Prochlorococcus	Prochlorococcus_HLII
**53b5e72c1f75d323de76ae2bfdb00f14**	Prochlorococcus	Prochlorococcus_HLI
**4c4e8ae3e6a400446b6bc086c49a9334**	Prochlorococcus	Prochlorococcus_HLI
**1401cfd6a11ecc38fc403120fd9baf85**	Prochlorococcus	Prochlorococcus_HLII
**93f178a72eea5198a7d679b5c07cfd6a**	Prochlorococcus	
**cfc527a99e1c21350a5ebef011e06412**	Prochlorococcus	Prochlorococcus_HLVI
**da0743ad03d541244793655fd973d050**	Prochlorococcus	Prochlorococcus_LLI
**8b5dd9350e443d05ae8a82e6c6e601ca**	Prochlorococcus	Prochlorococcus_HLI
**d8a85737429d1e36e5f90957de90847d**	Prochlorococcus	Prochlorococcus_HLI
**3f75af9bba716b458ed2573d8ed7f5cc**	Prochlorococcus	Prochlorococcus_LLI
**de41965b24dc5f62aa674f369e15572a**	Prochlorococcus	
**2963c726c22b74f8348b7eb9c6e31afc**	Prochlorococcus	Prochlorococcus_LLI
**8464d12a80902b6acae02e96859704fd**	Prochlorococcus	Prochlorococcus_HLI
**c28a1db474603a1b2606fdc5bd5015f3**	Prochlorococcus	
**fec52efff7ad2aaa25f9943ef3ba438b**	Prochlorococcus	
**8b50652a0bfb4bee2e23bb51bf97f672**	Prochlorococcus	
**5d1eca5eed8bc208c96c46d29f236b48**	Prochlorococcus	
**fa8f56301d871fa3b051479131fd3e4c**	Prochlorococcus	Prochlorococcus_HLII
**8b92f68324106fad581f3ef3712d335d**	Prochlorococcus	
**1d2cac6db3776ba1a62b44be50f94dd7**	Prochlorococcus	
**69aa85e864d5e524f76a213d43b23f8b**	Prochlorococcus	
**b5132cb54303b84bde6e8d69a65453c7**	Prochlorococcus	
**9e786b1cbaa09a1fcc973e0ca29eebaa**	Prochlorococcus	Prochlorococcus_LLI
**b6a2f0ad13c677813e025bbd13a559ec**	Prochlorococcus	

**69b4f1c82f576b1dc949a433edc72965**	Prochlorococcus	
**7c5342052300b4d9ccedbea55022eb3c**	Prochlorococcus	
**82f9feb736d6cc882d078513bb822de6**	Prochlorococcus	
**1b3de43da43a9830fdeab8bb23a7c161**	Prochlorococcus	
**4038a6aaac2e097a72180f062c7f4bb9**	Prochlorococcus	Prochlorococcus_HLI
**998029e58241e6de5f1cdec2dd0b12f3**	Prochlorococcus	
**3702e7f1c25acb47579f96204355c2ed**	Prochlorococcus	
**41fbdad0c1735ce7b427e645e700072e**	Prochlorococcus	Prochlorococcus_LLI
**c6fd8c9594bab36ae66c5f9ab9bcff10**	Prochlorococcus	
**efe3db31f261f2d2e3afe6f2a88105fd**	Prochlorococcus	
**7842b86580f66bd1572567e608e2eebe**	Prochlorococcus	
**fbddccb587f8cb799fe2a97e3a149a0a**	Prochlorococcus	
**a4e3ff368afe7c56c1d975c7fe09071e**	Prochlorococcus	Prochlorococcus_LLI
**2a247979fec2c96b842794e325c19108**	Prochlorococcus	
**a9fb0c63b927a17882c9a04069449cb9**	Prochlorococcus	
**6710fd74d0cf5cb2cde0f1c2b59b7702**	Prochlorococcus	
**9b643fd8ba4d61672ce9b4cde1891ee1**	Prochlorococcus	
**adea3bc1180a173251c5e8f2e9b9f347**	Prochlorococcus	
**1c4ab4ffbaa260ac964a349a099013dc**	Prochlorococcus	
**3ae353a1dbb78241b6cb18d9d6a6d1a4**	Prochlorococcus	
**8a2c57a22556a6364cdd3bed4ec632d2**	Prochlorococcus	
**416af87ddc1085cdc291a167d72b593d**	Prochlorococcus	
**4d5acf50d3d6f8d6be97acac2ea8d69d**	Prochlorococcus	
**6f364d7cc537d4ab60f9481c6baa2779**	Prochlorococcus	Prochlorococcus_HLI
**e89291e039fef4bfa12982a64146c5e0**	Prochlorococcus	
**84c1fe7acde454ec4a993d5786c299b8**	Prochlorococcus	
**a4ae6f1d56d8d96e4eec709113cd5a55**	Prochlorococcus	
**1e83339a0ba994bce28c975d9837fa25**	Prochlorococcus	
**005b9569c680ce3e57c50f45193e1534**	Prochlorococcus	
**8b7ec871c6dc23b79ce0ca416a67a52d**	Prochlorococcus	
**62742d17b1b8cfb87c2767d4b13e8034**	Prochlorococcus	
**6cc0da30dd839176d0557a2f06cb8d58**	Prochlorococcus	
**a3fa32e13c078d25fda23bd3e560b467**	Prochlorococcus	
**211b6158dbd950ea65f8556e1c59fcec**	Prochlorococcus	Prochlorococcus_HLII
**7362246952030f58b7eea37986e2cfd6**	Prochlorococcus	
**0a50f910c534e42d7493663bc98e762b**	Prochlorococcus	
**26890e84294dff1f0c41306271cab529**	Prochlorococcus	
**6813d0bbafc0b21360c73c9cbaa50bf4**	Prochlorococcus	
**0270299d39fc388fc903d55e3bb76c81**	Prochlorococcus	

**0d098828d4d393caa5a5d96ffb7b2730**	Prochlorococcus	
**2b572102bd80d619cde0da8b43d92342**	Prochlorococcus	
**6c9206afb01d77be1186ebfbf3c80901**	Prochlorococcus	
**b7eaae0e98746e7adf2257d9747c3fe2**	Prochlorococcus	
**62700af28d503dabf10ab732e805447f**	Prochlorococcus	
**5efcc204f0f02de0793ce2af2b3ac8f4**	Prochlorococcus	
**e57cc63c85075479bde22191b82a9153**	Prochlorococcus	
**b5d6843765969c231890371baecb938e**	Prochlorococcus	Prochlorococcus_LL
**ab92c278116ae7ca80b975b6414b8eac**	Prochlorococcus	
**5d470ea78b989aafe340256bae723040**	Prochlorococcus	
**f0339d822db49b1de112345c495d092a**	Prochlorococcus	
**2757833330790738c0705b67e9f9c872**	Prochlorococcus	
**bde39435b38913d48488e7e338a8c613**	Prochlorococcus	
**f3f4a4a5ad313aecafafa46ddb039d40**	Prochlorococcus	
**0cbaaad637e8d5c3c9c1b8db0837a898**	Prochlorococcus	Prochlorococcus_HLI
**56f59fec68838de246e2abeb3fd6c4b3**	Prochlorococcus	
**d6c84883346c2f088ecfe6a58046e470**	Prochlorococcus	
**1763c86c427d6e02f9c8222b5da30c83**	Prochlorococcus	
**1917b053f42abb814585f03872264e5f**	Prochlorococcus	
**729fc1bcf88364fc5128a0a011ef83cc**	Prochlorococcus	
**615a19ec81ec0493ea4c9fe0c9047e72**	Prochlorococcus	
**3bc551db62ef7581a9f655edc894b279**	Prochlorococcus	
**c5a3e50c014300c7a72628fcd6f7c6cb**	Prochlorococcus	
**4af6d1e2dd91ba6eed84c2cfe19ce939**	Prochlorococcus	
**2cc9291b57693a030533e40ebee044c1**	Prochlorococcus	
**e2db2bb24a2a014fd3873bd804d4f5f7**	Prochlorococcus	
**9dcdaa4c7f6011088f2ff586e8bde272**	Prochlorococcus	Prochlorococcus_HL
**f90f153638417372b797829360299230**	Prochlorococcus	
**ffc9b45ebc1f2389a7970d16c9efa434**	Prochlorococcus	
**2fe9c29e122d97078f1b5a16a85cb261**	Prochlorococcus	
**80707d13bda0527b5be13ea0ead41cd3**	Prochlorococcus	
**bd05a08a39f942436a01c63067787753**	Prochlorococcus	
**3f21e97d20a946285cfe1f0232a7e0d7**	Prochlorococcus	
**1c1c5585f76e947a9d168740634b0488**	Prochlorococcus	Prochlorococcus_HL
**f559262d3b614c709b022f7f3ead5221**	Prochlorococcus	
**9322fd3fc9e41132a020b7be4635cb2d**	Prochlorococcus	
**41655ea1ccf7370158034cd5322cd7d1**	Prochlorococcus	
**d1179fbc67887aeedbd8335fba750744**	Prochlorococcus	
**0ba6d10e147927d2d1e14622c6fbd435**	Prochlorococcus	

**421b608bd9127f78b2aaf5c2b958dda4**	Prochlorococcus	
**402f85fde91cd69b67f6734779461478**	Prochlorococcus	
**2aa1f5c7f4bc0b12b8b93ae888d3258d**	Prochlorococcus	
**36518b360f4a4356dad6a8b2623d698f**	Prochlorococcus	
**d48047c8692145dc3491d471c23f7248**	Prochlorococcus	
**6a81687222f7ec45f09f78a51350a7d5**	Prochlorococcus	
**6e19926d12bbb354819ab48b846b12b2**	Prochlorococcus	
**a23df6dfa6487f0d67868e989644c2c1**	Prochlorococcus	
**1758e38a3fa9850290bdaa85a9bf860e**	Prochlorococcus	
**7f8618340667e6488e248e774bd1340c**	Prochlorococcus	
**f007059ed347451318acf18d0721e3de**	Prochlorococcus	
**f1caa07ccd8e5d0efcbc6d500edc6ace**	Prochlorococcus	
**390c9d8d7ab94def9676e1f049dd46cf**	Prochlorococcus	
**b01784b07b162aa3e3b8a11e8dd11ea1**	Prochlorococcus	
**ee25628f222e43dd7c841674ccf6affb**	Prochlorococcus	
**c3cec8ce0c27e7a066ade4136c8b46c1**	Prochlorococcus	
**3e0682e8b31b484e27159dbccb3563c8**	Prochlorococcus	
**8b65681413c00d2d6f28ff3c59c33f1e**	Prochlorococcus	
**411b6dce4fcdbf9887f7881646a8574a**	Prochlorococcus	
**c4b967ee2a021aa0d259ea45c7a54cfa**	Prochlorococcus	
**564e263695c409bb8b2235201b046fd9**	Prochlorococcus	
**5cb4dd17578fabc31d45c84000659631**	Prochlorococcus	
**a85b8e7058cfca31bb224c3db5e33a16**	Prochlorococcus	
**eeb25c48bcb250fabc0d403561016fdf**	Prochlorococcus	
**2a1c8e0e890c366c4ddb158b7d88a674**	Prochlorococcus	
**ddf602a8e4bc01cf8f21c85fb6d1e8e0**	Prochlorococcus	
**b6499722c670cbdb0daeafcf173fa136**	Prochlorococcus	
**a84dba5828f4adc0c5ade67d41d09587**	Prochlorococcus	
**c8c68736d2587ce4e3afb0c75bb1eb3d**	Prochlorococcus	
**bae4a4d635aaad85a83d45e5477201fe**	Prochlorococcus	
**e7ea49f0cbd201601fed7d9630b82c1c**	Prochlorococcus	
**794032e05df482746f71b9379cd05da5**	Prochlorococcus	
**5ec1600da6bae172bbc8841ad411409d**	Prochlorococcus	
**8ddde688064656d98b75e9a94e56facf**	Prochlorococcus	
**a7415f4d62486f02bb0bf4fb56381e1c**	Prochlorococcus	
**2c99545fee80203496ed69aff76feddc**	Prochlorococcus	
**cabc7404556ddb8ce5eb0ef115c0b421**	Prochlorococcus	
**09c2e41276eeda31ea7ee956b4a96bcb**	Prochlorococcus	
**e945de40b7009c66ef4a603497de8fd0**	Prochlorococcus	

**7914f2cb180e56767913c618e10584ca**	Prochlorococcus	
**4cab0df7abb88b31989680d38a6e8df1**	Synechococcus	Synechococcus
**e94c43a6a4852fa204461351fbaaa24a**	Synechococcus	Synechococcus
**5f9fb096170d099dca2f6cea2eff2f77**	Synechococcus	
**6fdd688da33b366b837859cad836462a**	Synechococcus	Synechococcus
**9bbdb9accefe3cf4f38fa0a237f15d43**	Synechococcus	Synechococcus
**b44d953b99fc8e44d7b46b8e98a869ed**	Synechococcus	Synechococcus
**871bca12eb637e0efe230a554913d755**	Synechococcus	Synechococcus
**81f782f82ca67c0172e91db88981aae0**	Synechococcus	
**b4bde5fabe33a7f864361530153f48ad**	Synechococcus	
**5e4991ef40a6bd0a406131bcbf789ecb**	Synechococcus	Synechococcus
**744321cb23193b693d85f7e8e0fd6b90**	Synechococcus	
**ffa0efb18683bf9e82c2b1c1238ac16d**	Synechococcus	
**ffe7c32a9ecbfcfa2c3c821239b00d71**	Synechococcus	
**62e152e155718bea2ed2ffdcf18aa00c**	Synechococcus	
**08364a15cb1a0eb1e26d292e097ed358**	Synechococcus	
**5e28b8269ae9f87d516c543c7b3026d2**	Synechococcus	
**ff503a1c8c5aeb7262595a2cae225924**	Synechococcus	
**9b9fb81f9211fc4e0993b1be885c1f63**	Synechococcus	
**6471745fb4643841b033c37c8462b376**	Synechococcus	
**805f32d93fd6f6420866230f31cc0a22**	Synechococcus	
**933f1d8daada484af931d6bfadbe5f69**	Synechococcus	
**12735196326817db0fb298ccc036081b**	Synechococcus	
**0678daff15191e8a9639ecb6a53805ed**	Synechococcus	
**ef24a02a4aa6581d622f022afaa1c922**	Synechococcus	
**90daadacffb5843e34553c7aaf2b73e9**	Synechococcus	
**59eb38460af8dc63c20c4ecb3250b9bc**	Synechococcus	
**f645e216cab3a18eb47d96cc3b2b0cf3**	Synechococcus	

Table S2. Values used to quantify the genomic standard 16S rRNA gene copies.

Internal standard	ATCC	$conc_s$* (ng/μL)	V_s (μL)	$gDNA_l$ (bp)	rrn_s	C_s*
Thermus thermophilus	BAA-163D-5	0.2152 – 0.2445	20	2143708	2	3207782 - 4227299
Deinococcus radiodurans	27340D-5	0.1273 – 0.1575	20	3279485	3	2158324 - 2669190
Blautia producta	13939D-5	0.1600 – 0.1890	20	6244976	5	2373503 - 2803395

*These values ranged depending on the DNA extraction batch.

Table S3. Amplicon sample time and location paired with matched FCM sample time and location. All matchup samples were collected within a 30km distance and correspond with Figure S1. Samples shaded in green are within a 20km distance corresponding with the Figures of the main text. Matchups in the darkest green are within a 10km distance and correspond with Figure S2.

Amplicon sample	Amplicon sample date (YYYY-MM-DD)	Amplicon sample time	Amplicon sample lat (°N)	Amplicon sample lon	Flow cytometry sample date (YYYY-MM-DD)	Flow cytometry sample time	Flow cytometry sample lat (°N)	Flow cytometry sample lon	**ΔDistance (km)**	ΔTime (hr)
**10**	11/20/21	1:20	30.1215	238.4522	11/20/21	1:18	30.125	238.4554	**0.4962**	0.0333
**18***	11/20/21	14:43	28.503	235.714	11/20/21	19:40	28.4361	235.5971	**13.6348**	4.9500
**18***	11/20/21	14:43	28.503	235.714	11/20/21	14:16	28.5566	235.8042	**10.6382**	0.4500
**19**	11/21/21	2:03	28.2847	235.3517	11/21/21	1:03	28.3333333	235.45	**11.0386**	1.0000
**24**	11/21/21	11:53	27.0992	233.374	11/21/21	14:24	27.0673	233.3261	**5.9220**	2.5167
**26**	11/21/21	20:35	26.652	232.6401	11/21/21	20:04	26.7215	232.7522	**13.5557**	0.5167
**29**	11/22/21	2:10	25.9711	231.5141	11/22/21	2:03	25.9839	231.5359	**2.6028**	0.1167
**35**	11/22/21	13:13	24.6388	229.34	11/22/21	14:26	24.4889	229.0952	**29.8452**	1.2167
**36**	11/22/21	15:27	24.369	228.907	11/22/21	14:26	24.4889	229.0952	**23.2547**	1.0167
**38**	11/22/21	19:13	23.906	228.154	11/22/21	20:09	23.7973	227.9763	**21.7413**	0.9333
**39**	11/22/21	21:12	23.6708	227.7761	11/22/21	20:09	23.7973	227.9763	**24.7616**	1.0500
**41**	11/23/21	1:25	23.1732	226.9761	11/23/21	2:12	23.0736	226.8152	**19.8340**	0.7833
**44**	11/23/21	3:15	22.943	226.61	11/23/21	2:12	23.0736	226.8152	**25.5338**	1.0500
**48**	11/23/21	13:02	21.7525	224.7086	11/23/21	15:01	21.7402	224.6867	**2.6432**	1.9833
**49**	11/23/21	20:30	21.523	224.3435	11/23/21	21:28	21.4008	224.1571	**23.5949**	0.9667
**50**	11/23/21	22:21	21.2948	223.9874	11/23/21	21:28	21.4008	224.1571	**21.1615**	0.8833
**52**	11/24/21	2:07	20.8288	223.2527	11/24/21	2:39	20.7605	223.1475	**13.3142**	0.5333
**57**	11/24/21	21:37	19.6384	221.3856	11/25/21	2:30	19.6514	221.4323	**5.0997**	4.8833
**58**	11/26/21	1:52	19.3508	221.2797	11/26/21	3:00	19.1842	221.0898	**27.2123**	1.1333
**59**	11/26/21	4:04	19.025	220.9163	11/26/21	3:00	19.1842	221.0898	**25.4105**	1.0667
**66**	11/26/21	18:28	17.6862	219.8291	11/26/21	15:07	17.6748	219.8385	**1.6120**	3.3500
**67**	11/26/21	20:58	17.282	219.9525	11/26/21	21:01	17.2504	219.9626	**3.6738**	0.0500
**70**	11/27/21	3:00	16.036	219.9977	11/27/21	2:57	16.0449	219.9977	**0.9896**	0.0500

*Two FCM samples were paired with the same amplicon sample and treated as one matchup with 6 FCM replicates.

IV. References

[1] Cain KR, Ribalet F, Armbrust, EV. Discrete Flow Cytometry of Underway Samples from Gradients 4 (2021) Using a BD Influx Cell Sorter (Version 1.0). Zenodo [Dataset]. 2022. doi:10.5281/zenodo.6558008

[2] Ballantyne KN, et al. DNA contamination minimisation – finding an effective cleaning method. Australian Journal of Forensic Sciences. 2015; 47(4):428-39. doi:10.1080/00450618.2015.1004195

[3] Gifford S, et al. Microbial niche diversification in the galapagos archipelago and its response to el niño. Frontiers in Microbiology. 2020; 11:575194. doi:10.3389/fmicb.2020.575194

[4] Boström KH, et al. Optimization of DNA extraction for quantitative marine bacterioplankton community analysis. Limnology and Oceanography: Methods. 2004; 2(11):365-373. doi:10.4319/lom.2004.2.365

[5] Manganelli M, et al. Major Role of Microbes in Carbon Fluxes during Austral Winter in the Southern Drake Passage. PLoS ONE. 2009; 4(9):e6941. doi:10.1371/journal.pone.0006941

[6] Signori CN, et al. Microbial diversity and community structure across environmental gradients in Bransfield Strait, Western Antarctic Peninsula. Frontiers in Microbiology. 2014; 5:647. doi:10.3389/fmicb.2014.00647

[7] McNichol J, et al. Evaluating and Improving Small Subunit rRNA PCR Primer Coverage for Bacteria, Archaea, and Eukaryotes Using Metagenomes from Global Ocean Surveys. mSystems. 2021; 6(3). doi:10.1128/mSystems.00565-21

[8] Yeh Y-C, et al. Comprehensive single-PCR 16S and 18S rRNA community analysis validated with mock communities, and estimation of sequencing bias against 18S. Environmental Microbiology. 2021; 23(6):3240-50. doi:10.1111/1462-2920.15553

[9] Kelly L, et al. ProPortal: a resource for integrated systems biology of Prochlorococcus and its phage. Nucleic Acids Research. 2012; 40(DI):D632-40. doi:10.1093/nar/gkr1022

[10] Parada AE, Needham DM, Fuhrman JA. Every base matters: assessing small subunit rRNA primers for marine microbiomes with mock communities, time series and global field samples. Environmental Microbiology. 2016; 18(5):1403-14. doi:10.1111/1462-2920.13023

[11] Stoeck, T, et al. Multiple marker parallel tag environmental DNA sequencing reveals a highly complex eukaryotic community in marine anoxic water. Mol Ecol. 2010; 19:21–31. doi:10.1111/j.1365-294X.2009.04480.x
